# Stevioside from *Stevia rebaudiana* Bertoni Increases Insulin Sensitivity in 3T3-L1 Adipocytes

**DOI:** 10.1155/2013/938081

**Published:** 2013-12-11

**Authors:** Nabilatul Hani Mohd-Radzman, Wan Iryani Wan Ismail, Siti Safura Jaapar, Zainah Adam, Aishah Adam

**Affiliations:** ^1^Faculty of Pharmacy, Universiti Teknologi MARA, Puncak Alam Campus, 42300 Bandar Puncak Alam, Selangor, Malaysia; ^2^Medical Technology Division, Malaysian Nuclear Agency, Bangi, 43000 Kajang, Selangor, Malaysia

## Abstract

Stevioside from *Stevia rebaudiana* has been reported to exert antihyperglycemic effects in both rat and human subjects. There have been few studies on these effects *in vitro*. In this paper, radioactive glucose uptake assay was implemented in order to assess improvements in insulin sensitivity in 3T3-L1 cells by elevation of glucose uptake following treatment with stevioside. Oil Red-O staining and MTT assay were utilized to confirm adipocyte differentiation and cell viability, respectively. Findings from this research showed a significant increase in absorbance values in mature adipocytes following Oil Red-O staining, confirming the differentiation process. Stevioside was noncytotoxic to 3T3-L1 cells as cell viability was reduced by a maximum of 17%, making it impossible to determine its IC_50_. Stevioside increased glucose uptake activities by 2.1 times (*p* < 0.001) in normal conditions and up to 4.4 times (*p* < 0.001) in insulin-resistant states. At times, this increase was higher than that seen in positive control group treated with rosiglitazone maleate, an antidiabetic agent. Expressions of pY20 and p-IRS1 which were measured via Western blot were improved by stevioside treatment. In conclusion, stevioside has direct effects on 3T3-L1 insulin sensitivity via increase in glucose uptake and enhanced expression of proteins involved in insulin-signalling pathway.

## 1. Introduction

Epidemic-level emergence of many noncommunicable diseases as a result of modern lifestyle and dietary habits is worrying. Insulin resistance has commonly been linked to metabolic syndromes resulting in type 2 diabetes mellitus, as well as obesity. In fact, it is purported to underlie the progression of type 2 diabetes [1] and in cases of burn trauma, hyperlipidaemia and cancer cachexia [2]. It is important to note that insulin resistance occurs in an impaired insulin-signalling pathway, indicative of patients with type 2 diabetes. In normal individuals with fully functioning insulin-signalling activities, insulin will be secreted once blood glucose levels are increased following intake of food, and will subsequently bind to its receptor. This binding action will lead to several stages of signalling and phosphorylation cascades, resulting in migration of glucose transporter 4 (GLUT4) from cytoplasm to cellular membrane to take up extracellular glucose. However, in a state of insulin resistance, these signalling activities and cascades are interrupted, blocking said migration of GLUT4, if not disrupting the protein's expression altogether [3]. Hence, a better understanding of these mechanisms will possibly lead to breakthroughs in unravelling the secrets of both insulin resistance and diabetes.

As is often the case, traditional communities use local herbs in their folk and traditional medicines for treating hyperglycaemia and diabetes. Among these is *Stevia rebaudiana* Bertoni, a perennial herb commonly grown in tropical and subtropical regions, specifically in South America and Asia. In recent years, Malaysians too have taken a particular interest in this herb as it has been promoted as a sweetening alternative to sucrose, beneficial specifically for those with obesity and diabetes. *Stevia rebaudiana* has little to no caloric value despite its sweetening abilities, thus will not jeopardise patients' blood glucose levels, while fulfilling their cravings for sweet food and drinks [4]. *Stevia rebaudiana* is sweet due to its constituents of steviol glycosides including stevioside, rebaudioside A and rebaudioside C [5]. Furthermore, previous reports showed this plant has antioxidant [6] and antihyperglycaemic [7] properties, increasing its potential for use in adjuvant management of diabetes mellitus and associated conditions. There has been little investigation into such assertions which has prompted this study to evaluate how stevioside can affect insulin sensitivity, particularly through observation of glucose uptake, and expression of proteins involved in insulin-signalling pathway at a cellular level through use of 3T3-L1 adipocytes.

## 2. Methods

### 2.1. Materials

3T3-L1 preadipocytes were commercially acquired from ATCC (American Type Culture Collections, USA). Chemicals, including stevioside, cell supplements, and media, were mostly purchased from Sigma-Aldrich Co. (Germany) and Lonza (USA). Ultima Gold LLT scintillation cocktail and 2-deoxy-[1-3H]-glucose were commercially obtained from Perkin Elmer (USA). The antidiabetic drug rosiglitazone maleate (AVANDIA) was bought from a local drugstore. **β**-Actin, p-IRS1, and pY20 primary antibodies, and donkey anti-goat and goat anti-rabbit horseradish peroxidase (HRP) conjugated secondary antibodies were purchased from Santa Cruz (USA).

### 2.2. Cell Culture and Differentiation

3T3-L1 preadipocytes were cultured in Dulbecco's Modified Eagle's Media (DMEM). Cells were later differentiated with supplements of insulin, dexamethasone (DMX), and 3-isobutyl-1-methyl-xanthine (IBMX) following the procedures described by Ahn et al. [8] and Ismail et al. [9]. In order to induce insulin resistance, cells were treated with tumour necrosis factor-**α** (TNF-**α**) at 1.0 ng/mL for 4 days prior to treatment.

### 2.3. Oil Red-O Staining

3T3-L1 cells were washed with phosphate-buffered saline (PBS) before being fixed with a solution of 10% formaldehyde in PBS. After overnight incubation, the solution was discarded and Oil Red-O dye was introduced to cells for 10 minutes at room temperature. Subsequently, excess dye was washed with de-ionised water. The stain was later eluted out with 100% isopropanol and measured spectrophotomerically at 520 nm.

### 2.4. Cell Viability Test

3T3-L1 adipocytes were cultured in 96-well plates and were pretreated with stevioside (25–300 *μ*M) for 14, 24, 48, and 72 hours. After pretreatment, the cells received 5 mg/mL 3-(4,5-dimethylthiazol-2-yl)-2,5-diphenyltetrazolium bromide (MTT) and were further incubated for 4 hours. The purple formazan crystals were then dissolved with dimethyl sulfoxide (DMSO) before their absorbance values were read on a microplate reader at 590 nm.

### 2.5. Glucose Uptake Assay

Glucose uptake levels by differentiated 3T3-L1 adipocytes were analysed using a method previously described by Adam et al. [10], with minor modifications. Briefly, 3T3-L1 adipocytes were differentiated on 12-well plates. For insulin-resistance studies, cells were initially induced to insulin resistance with TNF-**α** treatment, as mentioned earlier. Cells were serum-starved for 2 hours. Next, they were washed with Krebs-Ringer bicarbonate (KRB) buffer and preincubated with a range of stevioside concentrations (30–150 *μ*M and 30–120 *μ*M) for half an hour, with or without insulin addition, with rosiglitazone as a positive control. 2-Deoxy-[1-3H]-glucose (1 *μ*Ci/mL) was then added to each well to initiate glucose uptake, and incubated for a further hour. Cells were then washed with ice-cold KRB buffer and solubilised by 0.1% sodium dodecyl sulphate (SDS). Finally, samples were collected and mixed with 15 mL of Ultima Gold LLT scintillation cocktail before being measured in a liquid scintillation counter.

### 2.6. Western Blotting

3T3-L1 cells were cultured and differentiated in 6-well plates. Cells were given TNF-**α** to induce insulin resistance, as described earlier, prior to treatment with stevioside. Treatments of stevioside and rosiglitazone maleate were given, in 60 and 90 *μ*M concentrations, for 24 hours. Cells were stimulated with insulin for 5 minutes prior to harvesting. From the preparation of cell lysates and loading of samples to Western blot analysis, procedures described by Ismail et al. [9] were followed, with minor modifications.

### 2.7. Statistical Analysis

Data were presented as mean ± standard error mean (SEM) for a given number of tests. The results were processed statistically by one-way analysis of variance (ANOVA) followed by Dunnett's post-hoc tests, using the Sigma Plot version 12 software. Statistically different means were recognised at *p* < 0.05.

## 3. Results

### 3.1. Oil Red-O Staining

**Figure 1:**
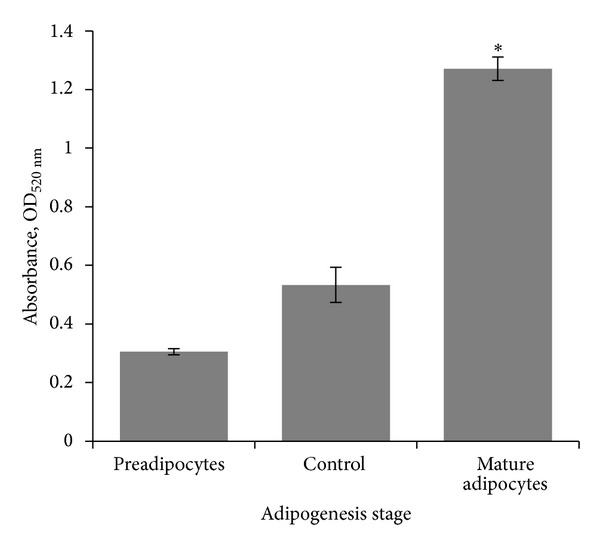
Effect of induction of differentiation on lipid accumulation in 3T3-L1 cells, presented by Oil Red-O staining. To quantify lipid accumulation in cells as a result of differentiation, the stain was eluted with 100% isopropanol and measured spectrophotometrically at 520 nm. Mean ± SEM (*n* = 3). *Significantly different from control (*p* < 0.05, ANOVA & Dunnett's test).

Effects of the supplements (insulin, DMX, and IBMX) on adipocyte differentiation are presented in Figure 1. In order to proceed with further experiments, adipocyte differentiation was confirmed by conducting the Oil Red-O staining procedure. Lipid stain from fully differentiated adipocytes was eluted out and measured quantitatively in a spectrophotometer, where the readings were found to be significantly increased when compared to control group.

### 3.2. MTT Cytotoxicity Test

**Figure 2:**
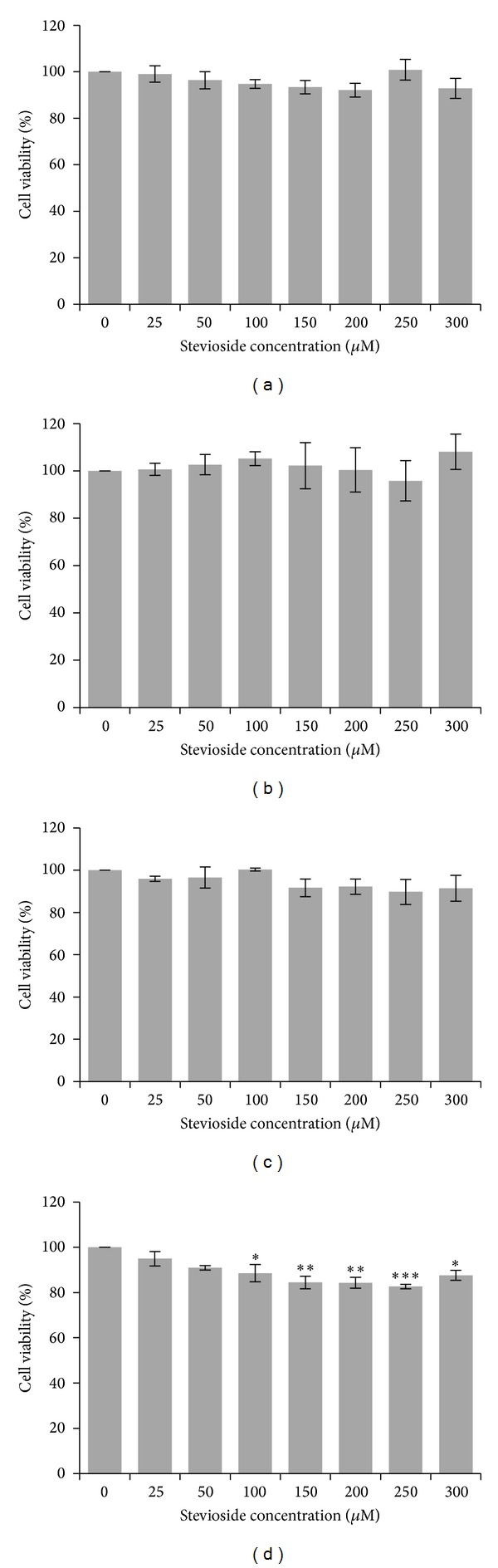
Viability of 3T3-L1 adipocytes treated with stevioside for (a) 14 hours, (b) 24 hours, (c) 48 hours, and (d) 72 hours. Mean ± SEM (*n* = 4). Statistically significant compared to control (*p < 0.05, **p < 0.01, and ***p < 0.001, ANOVA & Dunnett's test).

Cells were previously differentiated to mature adipocytes before treatment with stevioside (25–300 *μ*M) for 14, 24, 48, and 72 hours. Cell viability was determined from absorbance readings at 590 nm, corresponding to formazan crystals formed by living cells (Figure 2). There were no significant differences in cell viabilities in any of the stevioside treatment groups, at any treatment period, with the exception of those treated for a period of 72 hours. Viability of cells treated with 250 *μ*M of stevioside for 72 hours was reduced by 17% at most. No half-maximal inhibitory concentration (IC_50_) was found, as cell viability did not fall lower than the 80% benchmark.

### 3.3. Glucose Uptake Assay

#### 3.3.1. Optimum Insulin Concentration

**Figure 3:**
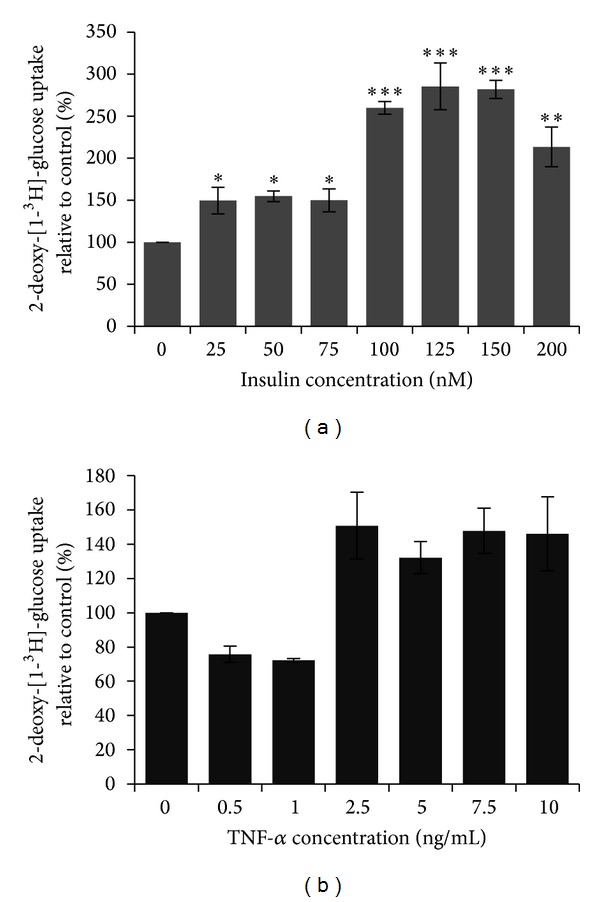
Glucose uptake in 3T3-L1 adipocytes exposed to (a) insulin and (b) TNF-*α*. Optimum insulin concentration to stimulate glucose uptake was 100 nM, while optimum TNF-*α* concentration to reduce glucose uptake was 1.0 ng/mL. Mean ± SEM (*n* = 4). Statistically significant compared to control (*p < 0.05, **p < 0.01, ***p < 0.001, ANOVA & Dunnett's test).

Prior to investigating effects of stevioside on glucose uptake in 3T3-L1 adipocytes, separate assays were conducted to find the optimum concentrations of insulin and TNF-**α** to be used. From Figure 3(a), it can be observed that starting from 100 nM of insulin, there was a highly significant increase in glucose uptake, with maximum uptake at 125 nM. There was no significant difference between these two groups. Therefore, 100 nM insulin was selected as the optimum concentration to be used for further experiments.

A separate glucose uptake assay was conducted to find the optimum TNF-**α** concentration for inducing an insulin-resistant state in 3T3-L1 adipocytes. TNF-**α** reduced glucose uptake in the cells up to a concentration of 1.0 ng/mL, beyond which there was an increase in glucose uptake at 2.5 ng/mL (Figure 3(b)). Since glucose uptake went down furthest at 1.0 ng/mL, this concentration was selected as the optimum TNF-**α** concentration to induce insulin resistance in cells.

#### 3.3.2. Glucose Uptake in Normal 3T3-L1 Adipocytes

**Figure 4:**
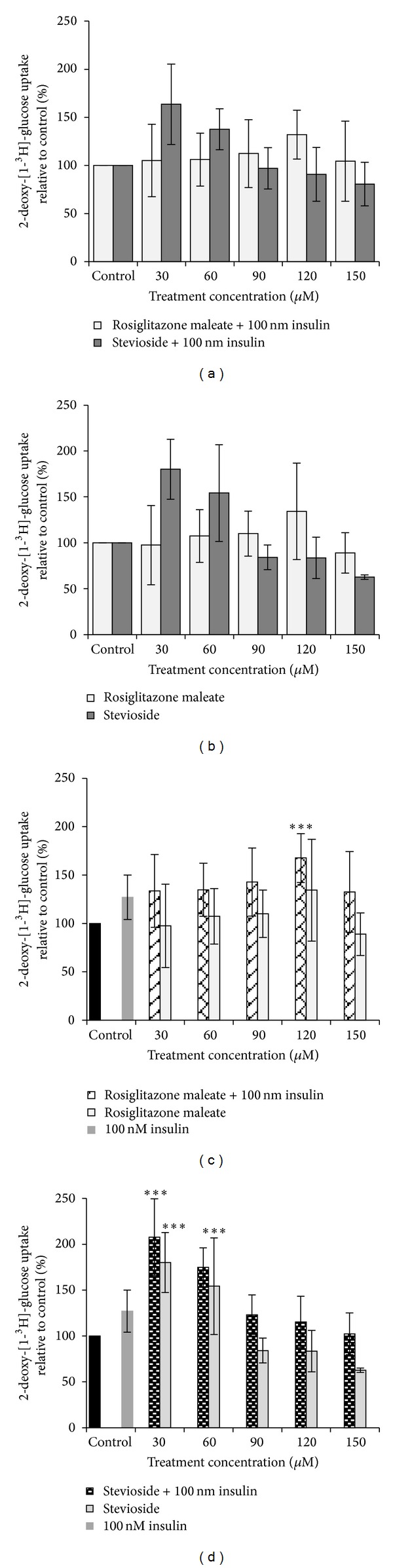
Effects of stevioside and rosiglitazone maleate on glucose uptake in normal 3T3-L1 adipocytes. (a) Treatment with stevioside or rosiglitazone maleate with insulin stimulation. (b) Treatment with stevioside or rosiglitazone maleate without insulin stimulation, (c) insulin-stimulated and non-insulin-stimulated groups treated with rosiglitazone maleate, and (d) insulin-stimulated and non-insulin-stimulated groups treated with stevioside. Mean ± SEM (*n* = 3). Statistically significant compared to control (***p < 0.001, ANOVA & Dunnett's test).

Glucose uptake assay was next conducted on normal, non-insulin-resistant adipocytes to study effects of stevioside. Glucose uptake by adipocytes was increased by treatment with stevioside or with rosiglitazone (Figure 4). Stevioside (30 *μ*M) elicited significant increase in glucose uptake, in both insulin-stimulated and non-insulin-stimulated groups. However, glucose uptake was higher in the insulin-stimulated group, by 2.1 times (*p* < 0.001) compared to control. This was better than what was observed in cells that were treated with rosiglitazone maleate, where glucose uptake in presence of insulin was increased by 1.7 times (*p* < 0.001) at a far higher concentration of 120 *μ*M.

#### 3.3.3. Glucose Uptake in TNF-*α* Induced Adipocytes

**Figure 5:**
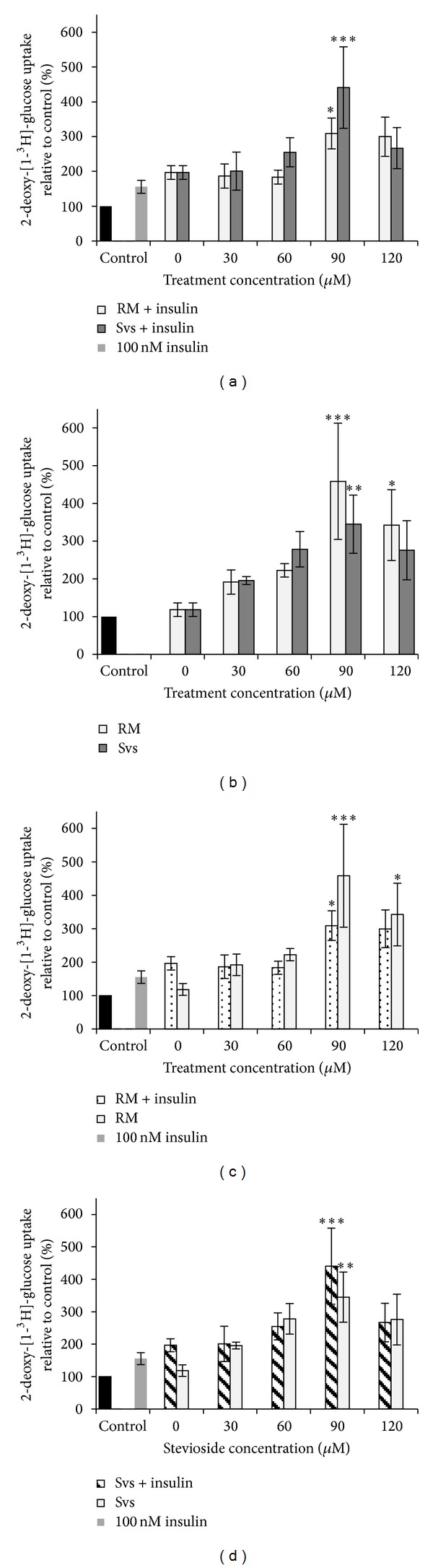
Effect of stevioside and rosiglitazone maleate on glucose uptake in TNF-**α** induced insulin-resistant 3T3-L1 adipocytes. (a) Treatment with stevioside or rosiglitazone maleate with insulin stimulation. (b) Treatment with stevioside or rosiglitazone maleate without insulin stimulation, (c) insulin-stimulated and non-insulin-stimulated groups treated with rosiglitazone maleate, and (d) insulin-stimulated and non-insulin-stimulated groups treated with stevioside. Mean ± SEM (*n* = 3). Statistically significant compared to control (*p < 0.05, **p < 0.01, ***p < 0.001, ANOVA & Dunnett's test). RM: rosiglitazone maleate, Svs: stevioside.

3T3-L1 cells were induced to insulin resistance using TNF-**α**, to test whether stevioside could exert similar effects on these cells as on normal adipocytes in terms of enhancing glucose uptake. Cells were pretreated with TNF-**α** prior to stevioside treatment and their glucose uptake was measured. In insulin-resistant adipocytes, stevioside was still able to stimulate glucose uptake although at a higher concentration of 90 *μ*M compared to the previous 30 *μ*M observed in normal adipocytes (Figure 5). Maximum increase in glucose uptake was observed in rosiglitazone-treated group (90 *μ*M) without insulin stimulation, with an elevation of up to 4.6 times (*p* < 0.001) when compared to control. This was followed by treatment with stevioside (90 *μ*M) with insulin stimulation, which elevated glucose uptake by 4.4-times (*p* < 0.001).

#### 3.3.4. Western Blotting

**Figure 6:**
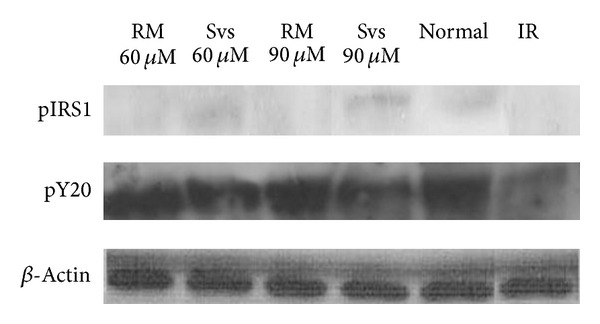
Band intensities observed via Western blotting, showing the different expression levels of phosphorylated insulin receptor substrate 1 (p-IRS1) and phosphorylated tyrosine (pY20), in groups treated with stevioside (Svs) and rosiglitazone maleate (RM) in comparison to the normal and insulin-resistant (IR) groups. **β*-*actin was used as a loading control. The experiment was repeated thrice.

In this study, expressions of two proteins (p-IRS1 and pY20) that are essential to the insulin-signalling pathway were measured by Western blotting technique. Both proteins were poorly expressed or not expressed at all in the insulin-resistant control group (Figure 6). Treatment with stevioside led to expression of both proteins while treatment with rosiglitazone elicited an increase in expression of pY20, though the effects of both treatments were hardly distinguishable from those observed in normal, non-insulin-resistant cells.

## 4. Discussion

Aim of this study was to investigate effects of stevioside on insulin sensitivity of 3T3-L1 cells. Oil Red-O is a common lipid stain used to colour lipid droplets *in vitro*, for quantification and for microscopic and imaging purposes. This specific staining method was used here to determine the degree to which adipocyte differentiation process (also called adipogenesis) had progressed since droplets secreted by adipocytes upon full differentiation to mature adipocytes will be stained (also referred to as lipid positive) [11]. Microscopic observation also revealed that lipid droplets were present in cytoplasm of fully differentiated adipocytes in abundance, compared to their absence in preadipocytes before the differentiation process.

As confirmation, there was a significant increase in absorbance values of eluted Oil Red-O stains from mature differentiated adipocytes (Figure 1). The stains from mature adipocytes were increased by 2.4 times (*p* < 0.05) when compared to control, which was the stain from empty wells; and were 4.2 times (*p* < 0.001) higher when compared to preadipocytes. This showed that the insulin-DMX-IBMX supplements combination was successful at hormonally triggering adipocyte differentiation in cells, most possibly via activation of peroxisome proliferator activator receptor *γ* (PPAR*γ*) and CCAAT/enhancer binding protein *α* (C/EBP*α*), as reported by Jin et al. [12].

Fully differentiated 3T3-L1 adipocytes were then subjected to MTT assay to determine effect of stevioside on cell morphology and viability. 3T3-L1 Cells were treated with stevioside at concentrations ranging from 25 to 300 *μ*M, for several different treatment periods: 14, 24, 48, and 72 hours. Treatment for 14 hours was done as it was the stipulated doubling time for 3T3-L1 adipocytes [13]. Viability of cells was not markedly altered by exposure to stevioside except at concentrations greater than 100 *μ*M following incubation for 72 hours. Even then cell viability was still higher than 80% with a maximum decrease of only 17%. Thus, median inhibitory concentration (IC_50_) could not be estimated from this study. Some slight, nonsignificant increase in cell viability of 3T3-L1 adipocytes was observed as has been previously reported [11, 14, 15]. It can be surmised that stevioside did not exert cytotoxicity on 3T3-L1 adipocytes.

To test effects of stevioside on insulin-sensitivity of 3T3-L1 adipocytes, its effects on glucose uptake by the adipocytes was determined. Radioactively labelled glucose uptake assays were conducted with use of antidiabetic agent, rosiglitazone as a positive control, as previously reported [10].

Prior to subjecting 3T3-L1 adipocytes to stevioside, response of the cells to insulin and to TNF-*α* were determined in separate assays to estimate optimum concentrations. These studies showed that glucose uptake was elevated with increase in insulin concentration in concentration-dependent manner reaching a plateau at 100 nM of insulin. This insulin concentration was taken as the optimum concentration for use in further glucose uptake assays using 3T3-L1 adipocytes as previously reported [16].

TNF-**α** has been implicated in the progression of insulin resistance; it disrupts phosphorylation of several proteins involved in insulin-signalling pathway, including IRS1, tyrosine, and most importantly, the downregulation of glucose transporter 4 (GLUT4) [3, 17]. TNF-**α** is also implicated in a number of catabolic states linked to insulin resistance such as sepsis and cancer even though its exact mechanism of actions remains unclear [18].

TNF-**α** caused a maximal decrease in glucose uptake at 1 ng/mL, beyond which glucose uptake was increased when compared to control. This observation contradicted initial expectation of a concentration-dependent decrease in glucose uptake by TNF-**α**, as was previously reported [19].

Normally differentiated, mature 3T3-L1 adipocytes which were not exposed to TNF-**α** were used to evaluate effect of stevioside on insulin sensitivity in normal, non-insulin-resistant state to ascertain effects of its usage in health. Our data showed that stevioside was better than the positive control, rosiglitazone, at increasing glucose uptake in normal adipocytes. Glucose uptake was maximally increased by 30 *μ*M of stevioside, both in presence or in absence of insulin stimulation, surpassing that of rosiglitazone at 120 *μ*M.

In TNF-*α* induced insulin resistance, glucose uptake was maximally increased by rosiglitazone or insulin at 90 *μ*M either in presence or absence of insulin. Since effect of rosiglitazone on glucose uptake was not different from that of stevioside, it may be concluded that both treaments were equally effective at enhancing glucose uptake in insulin-resistant cells.

There appeared to be interaction between rosiglitazone and insulin in the insulin-resistant state with regards to glucose-uptake, which was lower in presence of 100 nM insulin compared to in its absence. This observation was not seen with stevioside as presence of insulin further elevated glucose uptake compared to its absence. A similar trend of enhanced glucose uptake by stevioside and rosiglitazone was observed in normal adipocytes. It may be assumed that stevioside's interaction with insulin was unaffected by insulin resistance unlike that of rosiglitazone except higher concentration of stevioside was needed to elicit significant increase in glucose uptake during insulin-resistant state compared to normal state. The different behaviour of insulin-stimulated versus non-insulin stimulated cells to rosiglitazone has important bearing in hyperinsulinaemic states which often occur in insulin resistance, though not always. It is possible that stevioside is suitable for use in hyperinsulinaemic states, which, besides diabetes, often occur in certain cancers [20].

This study investigated two proteins—the phosphorylated insulin receptor substrate 1 (p-IRS1), and phosphorylated tyrosine (pY20) that act upstream of the insulin-signalling pathway. Our data showed both proteins were poorly expressed or not expressed at all in insulin-resistant control group, which imply disruptions in the insulin-signalling pathway as previously reported by Solomon et al. [17]. Such disruptions were successfully counteracted by stevioside which restored expression of both proteins, while rosiglitazone was also seen to increased expression of pY20 in insulin-resistant cells. Expression of pY20 was elevated by treatment with rosiglitazone or stevioside (60 *μ*M & 90 *μ*M) which provided support for potential ability of stevioside at sensitising cells to insulin signalling. p-IRS1 expression was observed in normal cells and in those treated with stevioside, although the bands were of low resolutions and intensities. No p-IRS1 bands were observed in insulin-resistant cells, including those treated with rosiglitazone. This showed that stevioside was able to restore p-IRS1 expressions, similar to those observed in normal group. Stevioside possibly have an effect upstream of the insulin-signalling pathway, by elevating levels of pY20 and probably p-IRS1 as well, which will result in GLUT4 translocation to increase uptake of extracellular glucose. Antihyperglycemic effects of stevioside on cell metabolism as a whole has also been previously reported [21].

## 5. Conclusion

Stevioside proved to be as effective as the antidiabetic agent, rosiglitazone, at enhancing glucose uptake in normal 3T3-L1 adipocytes or in cells induced to insulin resistance via exposure to TNF-*α*. In normal adipocytes, stevioside at a concentration that was a quarter that of rosiglitazone produced greater increase in glucose uptake than the antidiabetic agent, both in absence or presence of insulin-stimulation. In insulin-resistant cells, stevioside was as effective as rosiglitazone as similar concentrations of both produced comparable stimulation of glucose uptake in absence or presence of insulin. Enhancement of glucose uptake by stevioside was accompanied by increased expression of p-IRS1 and pY20, denoting involvement of GLUT-4 translocation. Further studies are needed to unravel mechanisms underlying use of stevioside in adjuvant management of diabetes mellitus and its associated complications.
