# Corrigendum to “Stevioside from *Stevia rebaudiana* Bertoni Increases Insulin Sensitivity in 3T3-L1 Adipocytes”

**DOI:** 10.1155/2016/2467420

**Published:** 2016-08-09

**Authors:** Nabilatul Hani Mohd-Radzman, Wan Iryani Wan Ismail, Siti Safura Jaapar, Zainah Adam, Aishah Adam

**Affiliations:** ^1^Faculty of Pharmacy, Universiti Teknologi MARA, Puncak Alam Campus, 42300 Bandar Puncak Alam, Selangor, Malaysia; ^2^Medical Technology Division, Malaysian Nuclear Agency, Bangi, 43000 Kajang, Selangor, Malaysia

The authors would like to indicate that there has been an inadvertent error in “Stevioside from* Stevia rebaudiana* Bertoni Increases Insulin Sensitivity in 3T3-L1 Adipocytes” [1].

 The Western blot image of Figure  6 that was uploaded for the *β*-actin protein bands is incorrect. The corrected image of the *β*-actin protein for Figure  6 is hereby attached. This error does not alter or affect the discussions and conclusions that have been reached in this paper.

Also in Figure  6, the pIRS1 bands were rearranged to match the pY20 group, because the blots were run in a different order.

## Figures and Tables

**Figure 6:**
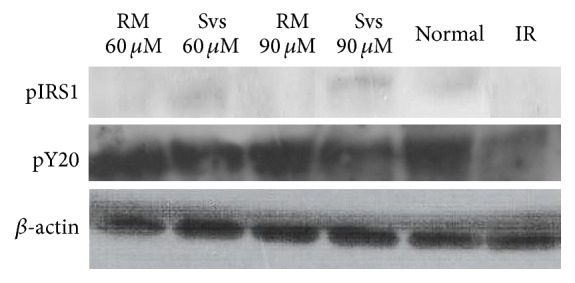
Band intensities observed via Western blotting, showing the different expression levels of phosphorylated insulin receptor substrate 1 (p-IRS1) and phosphorylated tyrosine (pY20), in groups treated with stevioside (Svs) and rosiglitazone maleate (RM) in comparison to the normal and insulin-resistant (IR) groups. *β*-actin was used as a loading control. The experiment was repeated thrice.
